# Supplementary material for: Molecular mechanism underlying the increased risk of colorectal cancer metastasis caused by single nucleotide polymorphisms in LI-cadherin gene
Source: Sci Rep. 2023 Apr 20;13:6493. doi: 10.1038/s41598-023-32444-4 (PMC10117238; doi:10.1038/s41598-023-32444-4)
Supplement: Supplementary file 1 — Supplementary Information 1. [file 41598_2023_32444_MOESM1_ESM.pdf]

Supplementary Information

Molecular mechanism underlying the increased risk of colorectal cancer metastasis caused by single nucleotide polymorphisms in LI-cadherin gene

Anna Yui[1], Daisuke Kuroda[2,3], Takahiro Maruno[4,5], Makoto Nakakido[1], Satoru Nagatoishi[6], Susumu Uchiyama[4], and Kouhei Tsumoto[1,6,7]

[1]Department of Bioengineering, School of Engineering, The University of Tokyo, 7-3-1, Hongo, Bunkyo-ku, Tokyo 113-8656, Japan
[2]Medical Device Development and Regulation Research Center, School of Engineering, The University of Tokyo, 7-3-1, Hongo, Bunkyo-ku, Tokyo 113-8656, Japan
[3]Research Center for Drug and Vaccine Development, National Institute of Infectious Diseases, 1-23-1 Toyama, Shinjuku-ku, Tokyo 162-8640, Japan
[4]Department of Biotechnology, Graduate School of Engineering, Osaka University, 2-1 Yamadaoka, Suita-shi, Osaka 565-0871, Japan
[5]U-Medico Inc., 2-1 Yamadaoka, Suita-shi, Osaka 565-0871, Japan
[6]Institute of Medical Science, The University of Tokyo, 4-6-1, Shirokanedai, Minato-ku, Tokyo 108-8639, Japan
[7]Department of Chemistry and Biotechnology, School of Engineering, The University of Tokyo, 7-3-1, Hongo, Bunkyo-ku, Tokyo 113-8656, Japan

*Corresponding author: Kouhei Tsumoto
E-mail: tsumoto@bioeng.t.u-tokyo.ac.jp

Table S1: Constructs of LI-cadherin used in this study.
Table S2: Percentage of trajectories from 40 to 400 ns of which the distance between Lys115-NZ or Glu115-OE1 and Lys117-NZ was 4 Å or less.

Figure S1: Verification of LI-cadherin expression in CHO cells.
Figure S2: RMSD values of Cα atoms during the simulations of EC1-4K115E homodimer.
Figure S3: Distance between the center of gravity of Ile169 or Leu171 in chain A and Ala373 or Phe376 or Ala400 in chain B during the simulations of the A. EC1-4WT homodimer or B. EC1-4K115E homodimer.
Figure S4: Distance between the center of gravity of Met173 in chain A and Ala400 or Met402 in chain B during the simulations of the A. EC1-4WT homodimer or B. EC1-4K115E homodimer.
Figure S5: Distance between the NZ atom of Lys117 and A. NZ atom of Lys115 in the EC1-4WT homodimer, B. OE1 atom, or C. OE2 atom of Glu115 in the EC1-4K115E homodimer.

Movie S1. K115Edimer-Run3
Movie S2. WTdimer-Run1

Table S1: Constructs of LI-cadherin used in this study.

Construct name	Sequence[c]	Usage
EC1-4WT[a]<br>EC1-4K115E[a]	23–441	SV-AUC, CD, and DSC
EC1-2WT[a]<br>EC1-2K115E[a]	23–236	
WT-CHO[b]<br>K115E-CHO[b]<br>E739A-CHO[b]<br>2mut-CHO[b]	23–832	Cell aggregation assays
EC1-4WT<br>EC1-4K115E	23–441	MD simulations

a. Myc-tag (EQKLISEEDL), NSAVD sequence, and 6xHis-tag were added to the C-termini of these sequences.

b. Gly-Ser linker and monomeric GFP were added to the C-terminus.

c: Residue numbering follows the numbering of Uniprot entry Q12864.

Table S2. Percentage of trajectories from 40 to 400 ns of which the distance between Lys115-NZ or Glu115-OE1 and Lys117-NZ was 4 Å or less.

	WT				K115E		
	Run 1	Run 2	Run 3		Run 1	Run 2	Run 3
Lys115-NZ (%)	0.00833	0.0222	0.00278	Glu115-OE1 (%)	25.0	27.5	26.0
				Glu115-OE2 (%)	24.3	29.0	26.2

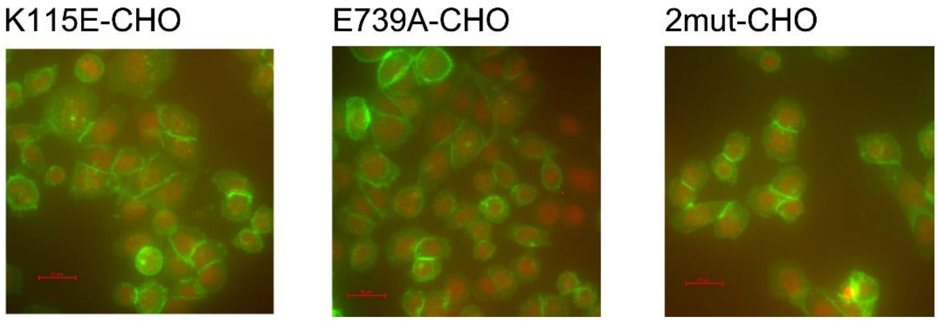

Figure S1. Verification of LI-cadherin expression in CHO cells. Expression of LI-cadherin on the cell surface was confirmed by observing the fluorescence of GFP with an In Cell Analyzer 2000 instrument. Fluorescence of GFP and Hoechst 33342 are indicated in green and red, respectively. Expression of LI-cadherin was observed in all constructs shown above. Scale bars represent 20 μm. LI-cadherin expression on the cell surface of WT-CHO was previously confirmed (9).

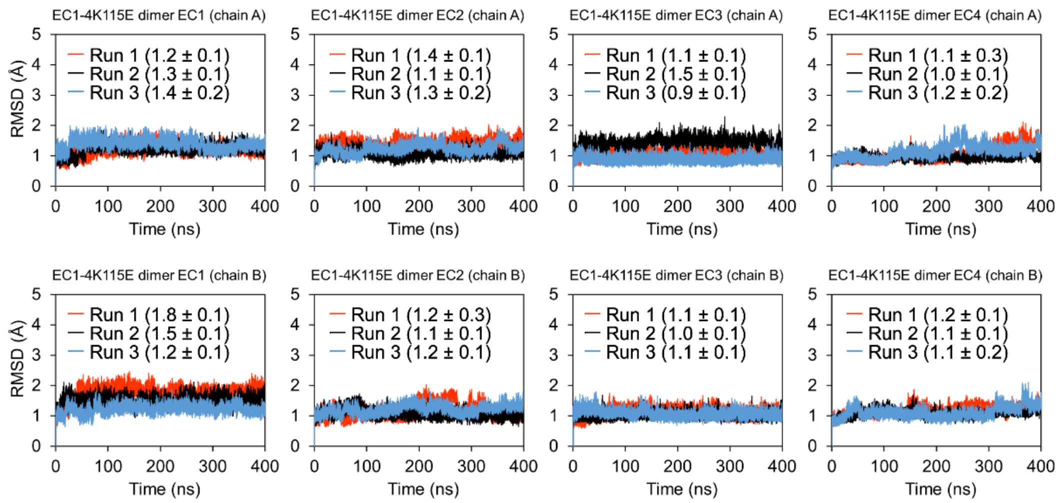

Figure S2. RMSD values of Cα atoms during the simulations of EC1-4K115E homodimer. As the molecule showed high flexibility at Ca^{2+}-free linker, RMSD values of each domain was calculated individually. Five Cα atoms at N-terminus were excluded from the calculation of RMSD of EC1 as they were disordered. Averages and standard deviations from 40 to 400 ns are shown in the graph in angstrom unit.

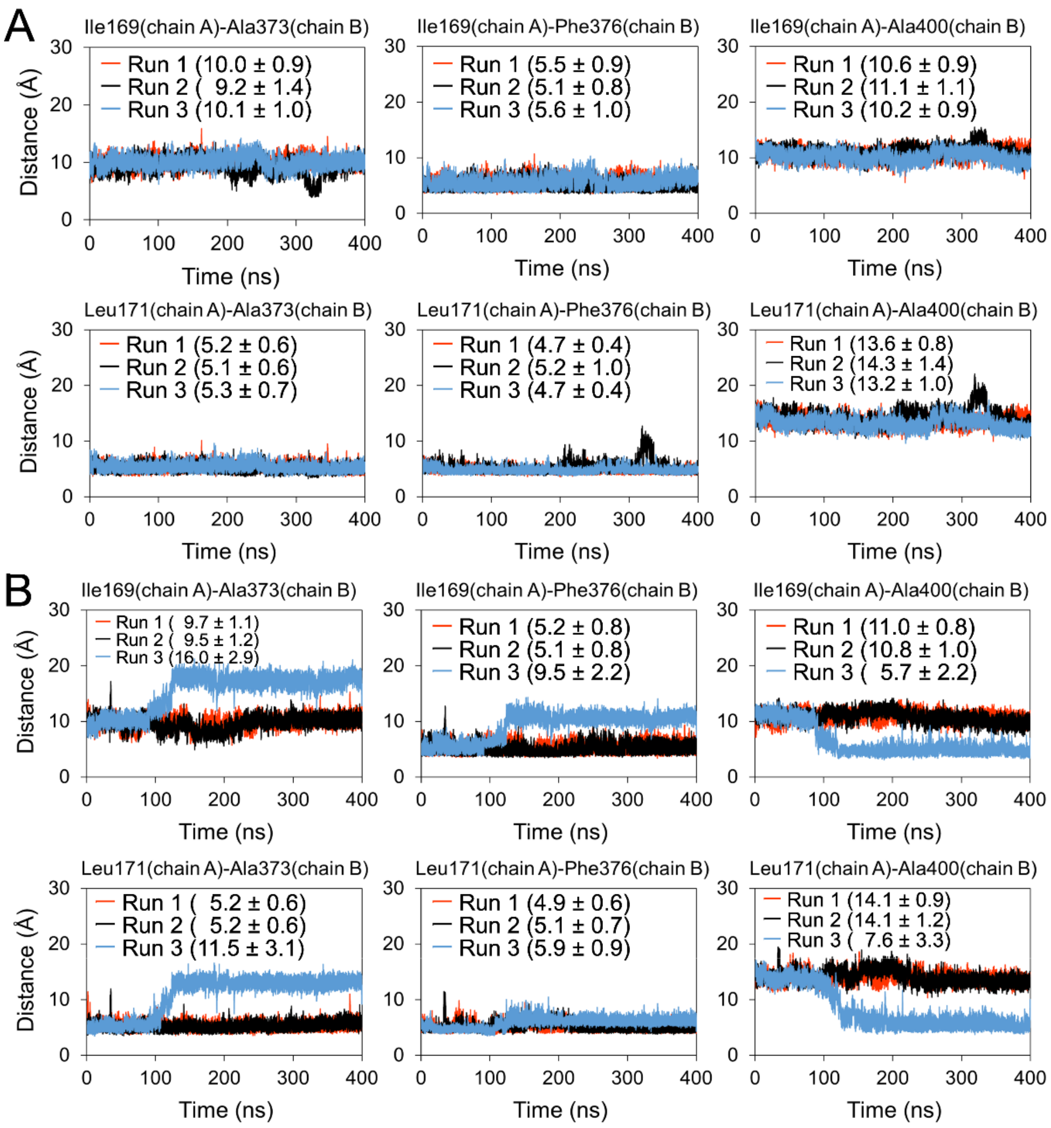

Figure S3. Distance between the center of gravity of Ile169 or Leu171 in chain A and Ala373 or Phe376 or Ala400 in chain B during the simulations of the **A.** EC1-4WT homodimer or **B.** EC1-4K115E homodimer. Only Run 3 of the simulations of EC1-4K115E homodimer showed a significant increase or decrease in the middle of the simulations. Averages and standard deviations from 40 to 400 ns of each simulation are shown in the graph in angstrom unit.

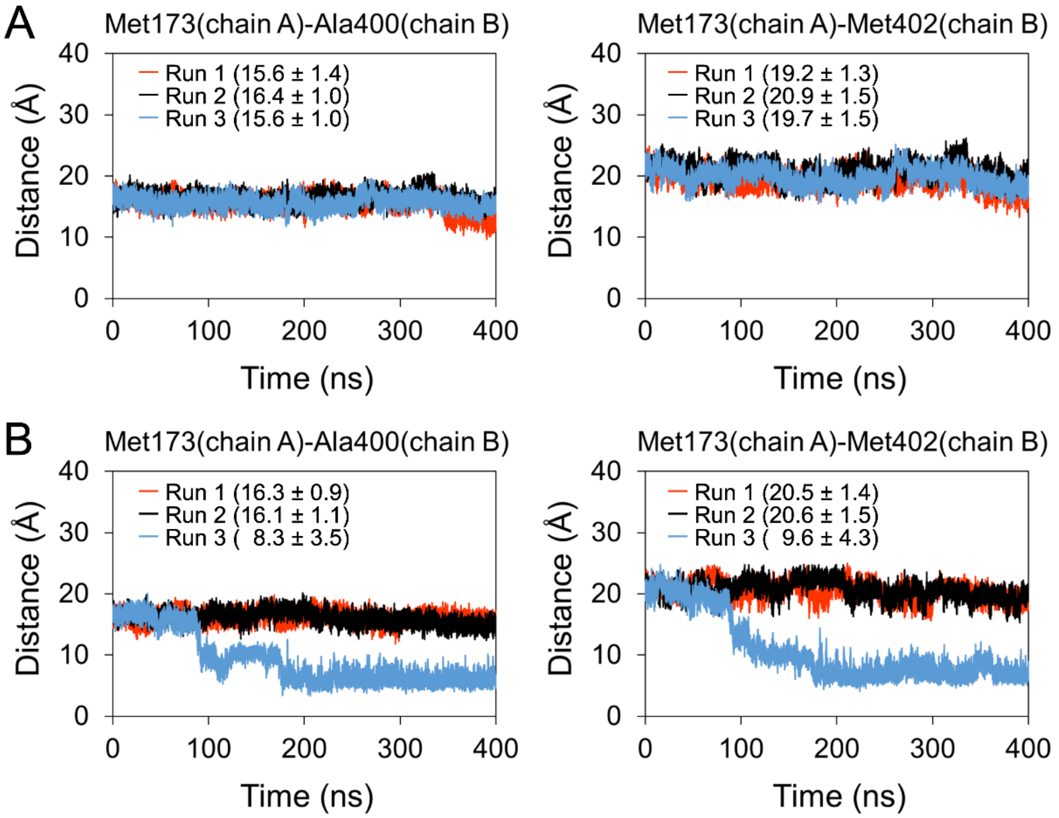

Figure S4. Distance between the center of gravity of Met173 in chain A and Ala400 or Met402 in chain B during the simulations of the **A.** EC1-4WT homodimer or **B.** EC1-4K115E homodimer. Only Run 3 of the EC1-4K115E homodimer showed a drastic decrease after approximately 100 ns. Averages and standard deviations from 40 to 400 ns of each simulation are shown in the graph in angstrom unit.

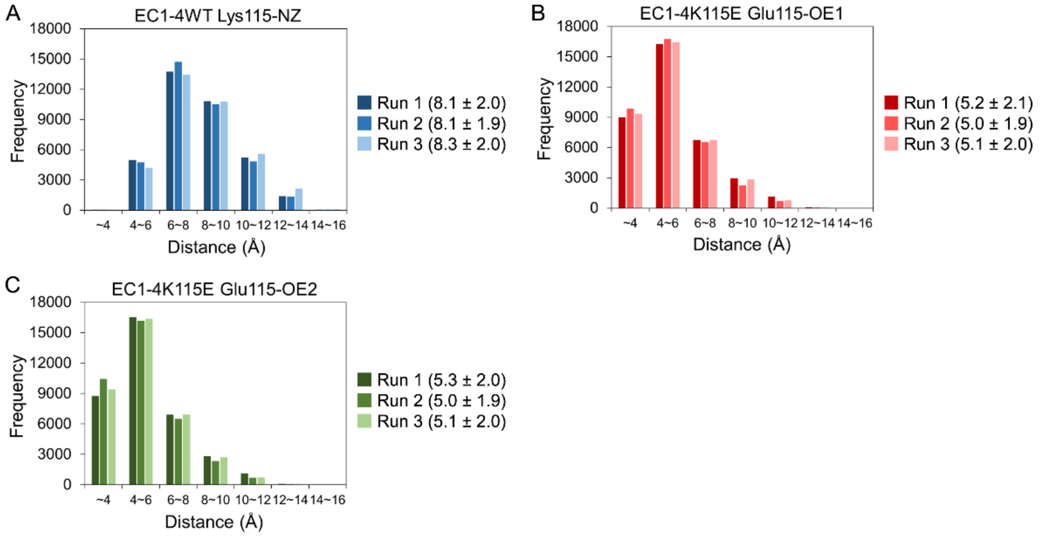

Figure S5. Distance between the NZ atom of Lys117 and **A.** NZ atom of Lys115 in the EC1-4WT homodimer, **B.** OE1 atom, or **C.** OE2 atom of Glu115 in the EC1-4K115E homodimer. All atoms belonged to chain A. Averages and standard deviations from 40 to 400 ns of each simulation are shown in the graph in angstrom unit.
